# Osmotic Stress Uncovers Correlations and Dissociations Between Larval Zebrafish Anxiety Endophenotypes

**DOI:** 10.3389/fnmol.2022.900223

**Published:** 2022-06-24

**Authors:** Ruey-Kuang Cheng, Jazlynn Xiu Min Tan, Kai Xin Chua, Cheryl Jia Xin Tan, Caroline Lei Wee

**Affiliations:** Institute of Molecular and Cell Biology, A*STAR, Singapore, Singapore

**Keywords:** zebrafish, stress, anxiety, osmotic stress, endophenotype, stress anorexia

## Abstract

Larval zebrafish are often used to model anxiety disorders. However, since it is impossible to recapitulate the full complexity and heterogeneity of anxiety in this model, examining component endophenotypes is key to dissecting the mechanisms underlying anxiety. While individual anxiety endophenotypes have been examined in zebrafish, an understanding of the relationships between them is still lacking. Here, we investigate the effects of osmotic stress on a range of anxiety endophenotypes such as thigmotaxis, dark avoidance, light-dark transitions, sleep, night startle, and locomotion. We also report a novel assay for stress-induced anorexia that extends and improves on previously reported food intake quantification methods. We show that acute <30 min osmotic stress decreases feeding but has no effect on dark avoidance. Further, acute osmotic stress dose-dependently increases thigmotaxis and freezing in a light/dark choice condition, but not uniform light environmental context. Prolonged >2 h osmotic stress has similar suppressive effects on feeding while also significantly increasing dark avoidance and sleep, with weaker effects on thigmotaxis and freezing. Notably, the correlations between anxiety endophenotypes were dependent on both salt and dark exposure, with increased dissociations at higher stressor intensities. Our results demonstrate context-dependent effects of osmotic stress on diverse anxiety endophenotypes, and highlight the importance of examining multiple endophenotypes in order to gain a more complete understanding of anxiety mechanisms.

## Introduction

Stress exerts profound effects on our health, both mentally and physically. Acute stress induces immediate adaptive responses such as activation of the hypothalamic-pituitary-adrenal (HPA) stress axis, whereas prolonged or severe exposure to stress could lead to maladaptive reactions and may cause long-term health problems, such as anxiety and eating disorders (Barton, 2002; Erhardt et al., 2006; Bose et al., 2009; Lucassen and Cizza, 2012). The mechanisms by which different degrees of stress and HPA axis activation influence emotional states and behavior remain to be completely established.

Zebrafish, as freshwater fish, are sensitive to environmental stressors such as the salinity of the water (Barton, 2002; De Marco et al., 2014). Importantly, they also share similar neuroanatomy and physiology, including the HPA stress response, with humans (Kalueff et al., 2014; Stewart et al., 2014, 2015). Multiple behavioral endophenotypes have been described in the literature on zebrafish stress and anxiety (Tan et al., 2022). Thigmotaxis, or the propensity to stay close to the wall of the testing arena, is thought to be a proxy of anxiety behavior in animals (Wagle et al., 2017). Anxiolytic drugs decrease the level of thigmotaxis while anxiogenic drugs enhance thigmotaxis in larval zebrafish (Richendrfer et al., 2012). Another proxy of anxiety is dark avoidance behavior in a light/dark (L/D) choice assay, and anxiolytic or anxiogenic drugs similarly affect dark avoidance in expected directions (Steenbergen et al., 2011). Additionally, locomotion during light-dark (L-D) transitions has been used to represent an anxiety endophenotype (Faught and Vijayan, 2022; Sveinsdóttir et al., 2022). The correlations or dissociations between these anxiety endophenotypes, as well as others such as sleep, startle, and feeding are not completely known and may vary according to stressor identity or intensity (Tan et al., 2022).

In this study, we utilized osmotic stress to tease apart the relationships between multiple anxiety endophenotypes. Salt clearly induces a stress response and HPA axis activation in freshwater fish such as zebrafish, as measured by cortisol release (Kammerer et al., 2010; Yeh et al., 2013). De Marco et al. (2014) showed that in larval zebrafish, sodium chloride (NaCl) exposure increased cortisol levels in a concentration-dependent manner, peaking at 5–10 min after exposure. Further, osmolarity is stable, can be quantified precisely, and can be uniformly maintained in the arena regardless of the fish’s location; these properties are more challenging to ensure when using other stressors such as acoustic, mechanical, or temperature manipulations.

We found that osmotic stress affected different anxiety endophenotypes in an intensity- and context-dependent manner. For example, dark avoidance and thigmotactic behaviors were dissociable – acute osmotic stress affected thigmotaxis when a dark zone was present but not in uniform light conditions, whereas prolonged osmotic stress significantly increased dark avoidance with weaker effects on thigmotaxis. Sleep was also reduced under prolonged salt stress, while L-D transitions and night-time acoustic startle were unaffected. Notably, dissociations between anxiety endophenotypes increased with stressor intensity. Building on previous work, we established a novel method of quantifying osmotic stress-induced anorexia in zebrafish, and found suppressive effects of both acute and prolonged osmotic stress on feeding. Such diverse effects of a single stressor on anxiety endophenotypes suggest distinct underlying mechanisms, adaptations, or coping strategies in response to varying degrees of stress exposure.

## Materials and Methods

### Subjects

Adult zebrafish were maintained on a 14:10-h light/dark cycle at 28°C in the Institute of Molecular and Cell Biology (IMCB) Zebrafish Facility. All protocols and procedures involving zebrafish were approved by the A*STAR Biomedical Research Council Institutional Animal Care and Use Committee (IACUC). Wild type zebrafish from AB background were used for sleep and locomotion and L/D choice assays. Nacre (*mitfa*−/−) and Tupfel Long-Fin (TL) fish were used for comparison of salt stress on feeding with wild-type AB fish.

### Breeding of Larval Zebrafish for Behavioral Testing

Embryos were bleached at the shield stage and raised in E3 medium in a 14:10-h light/dark cycle at 28°C. Fish were raised at a density of 250 fish per petri dish (15 cm diameter) and fed an excess of paramecia daily from 5 dpf. Dead embryos were removed and E3 medium was changed on a daily basis.

### Salt Stress Manipulations

Sodium chloride (5 M stock solution) was diluted in sterile water to obtain concentrations of 0.5, 1, 1.5, 2, and 2.5 M. Final test volumes were 30 mL for feeding assays, 3 mL for L/D choice and 300 μL for sleep assays. Zebrafish survive overnight in 50 and 100 mM salt, however 150 mM salt solution is lethal after 2–4 h. Hence, while 50, 100, and 150 mM (and higher in the case of feeding) were utilized in acute assays, a maximum of 100 mM salt was used in prolonged exposure conditions (feeding, L/D choice, sleep and locomotion assays). For the L/D choice assay, fish were incubated in E3 or salt solution either from the beginning of the 10 min acclimatization period (acute exposure) or for 2–4 h prior to testing (prolonged exposure). For the sleep and locomotion assay, fish were incubated for the entire recording time. For feeding assays, they were exposed acutely (10 min prior to feeding) or for 2 h prior to feeding (from the start of the starvation period). By using an osmometer (VAPRO-5520), the osmolarity reading of our salt concentrations are 1.5 ± 1.5 mM/kg (0 mM), 85 ± 6 mmol/kg (50 mM), and 183 ± 4 mmol/kg (100 mM, *n* = 2 measurements for all concentrations).

### Light/Dark (L/D) Choice Assay

**FIGURE 1:**
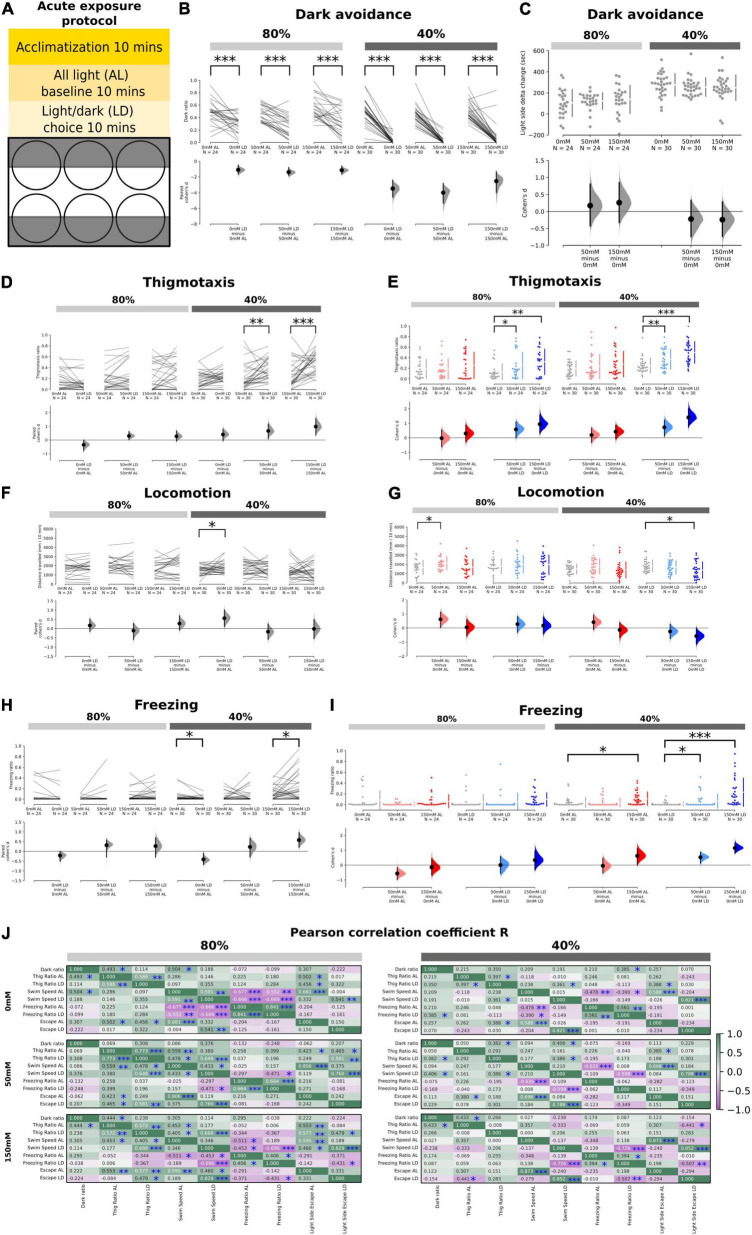
Effects of acute salt stress on thigmotaxis and dark avoidance. **(A)** Schematic showing the experimental procedure and the setup of the 6-well plate for the light/dark choice assay. **(B)** Ratio of time spent in the “dark half” of the chamber in the AL and LD condition across salt concentrations (80%: *p* = 0.0006 in 0 mM; *p* < 0.0001 in 50 mM; *p* = 0.0004 in 150 mM, 40%: *p* < 0.0001 in 0, 50, and 150 mM). **(C)** No significant delta change of time spent in the light side between the AL and LD period in 50 – 0 mM (80%: *p* = 0.542, 40%: *p* = 0.394) and 150 – 0 mM (80%: *p* = 0.366, 40%: *p* = 0.361) salt concentrations at both 80 and 40% transparency. Positive numbers mean an increase of time spent on the light side (i.e., dark avoidance). **(D)** Thigmotaxis ratio between AL and LD conditions across salt concentrations. Significant increases were observed at 50 mM (*p* = 0.004) and 150 mM (*p* < 0.0001) salt concentrations at 40% transparency. **(E)** Thigmotaxis ratio between AL and LD periods as a function of salt concentrations (*p* = 0.047 for interactions in 40% and *p* = 0.028 for interactions in 80%, two-way mixed ANOVA). Significant increases were observed in the LD condition at both 80 and 40% transparency (80%: *p* = 0.0468 in 50 – 0 mM; *p* = 0.0016 in 150 – 0 mM, 40%: *p* = 0.007 in 50 – 0 mM; and *p* < 0.0001 150 – 0 mM) but not in the AL condition. **(F)** Total distance traveled between AL and LD conditions across salt concentrations with significant increase (*p* = 0.0448) in 0 mM AL vs. LD for 40% transparency. **(G)** A significant increase in locomotion was observed at 50 mM salt concentration in the AL condition at 80% transparency (*p* = 0.0356) and at 150 mM in the LD condition at 40% transparency (*p* = 0.0282). **(H)** The freezing ratio was significantly lower in the LD condition compared to the AL condition at 40% transparency in the control group (*p* = 0.0306) but significantly higher in the 150 mM salt concentration group (*p* = 0.013). **(I)** The freezing ratio was significantly increased at 150 mM salt concentration in the AL condition at 40% transparency (*p* = 0.0168) and at all salt concentrations in the LD condition at 40% transparency (50 – 0 mM: *p* = 0.0322, 150 – 0 mM: *p* < 0.0001). **(J)** Correlation coefficient (*r*) of the various endophenotype measurements during the AL and LD period in different salt concentrations at both 40 and 80% transparency (*p < 0.05, ^**^p < 0.01, ^***^p < 0.001).

**FIGURE 2:**
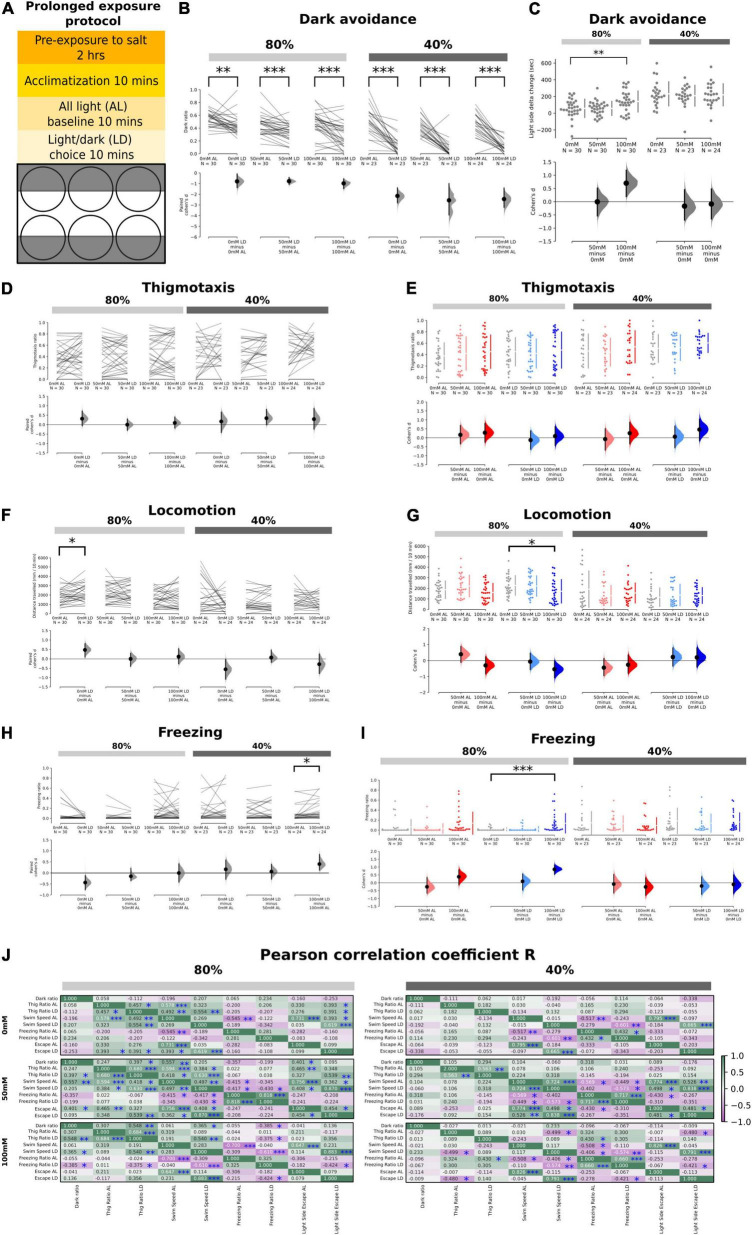
Effects of prolonged exposure to salt stress on thigmotaxis and dark avoidance. **(A)** Schematic showing the experimental procedure and the setup of the 6-well plate for the light/dark choice assay with prolonged exposure to salt stress. **(B)** Ratio of time spent in the “dark half” of the chamber in the AL and LD condition across salt concentrations (80%: *p* = 2.6 × 10^– 3^ in 0 mM; *p* = 4.0 × 10^– 4^ in 50 mM, and *p* = 4.0 × 10^– 4^ in 100 mM, 40%: *p* < 0.0001 in 0, 50, and 100 mM). **(C)** Significant delta change of time spent in the light side between the AL and 80% transparency LD period in 100 – 0 mM (*p* = 0.0098) but not 50 – 0 mM (*p* = 0.987) salt concentrations. No significant changes for AL vs. 40% transparency LD (*p* = 0.581 for 50 – 0 mM, *p* = 0.776 for 100 – 0 mM). Positive numbers mean an increase of time spent on the light side (i.e., dark avoidance). **(D,E)** No significant changes in thigmotaxis ratios in both 80 and 40% transparency across both AL and LD conditions. **(F)** A significant increase in locomotion was observed in the control group at 80% transparency in the LD condition compared to the AL condition (*p* = 0.0156). **(G)** A significant reduction in locomotion was observed at 100 mM at 80% transparency in the LD condition (*p* = 0.0374). **(H)** The freezing ratio was significantly higher in the LD condition compared to the AL condition at 40% transparency (*p* = 0.01). **(I)** The freezing ratio was significantly increased at 100 mM salt concentration in the LD condition at 80% transparency (*p* < 0.0001). **(J)** Correlation coefficient (*r*) of the various endophenotype measurements during the AL and LD period in different salt concentrations at both 40 and 80% transparency (*p < 0.05, ^**^p < 0.01, and ^***^p < 0.001).

The details of this assay were recently published and described in Basnakova et al. (2021). For this study we modified the Python (v3.9) scripts to test six fish simultaneously using a 6-well plate (see Figures 1A, 2A). The 6-well plate was placed on top of an Apple iPad Air and images were generated and projected using Microsoft Powerpoint, with the dark half of the arena at either 40 or 80% transparency. Fish were placed in this 6-well plate first for 10 min for acclimatization with the designated salt concentration in the wells in an all-light (AL) condition. For prolonged exposure, 20 fish were incubated in E3 or salt solution in a 10-cm dish for 2–4 h before being placed in the 6-well plate. Following the acclimatization, another 10 min were recorded as the baseline AL condition. Subsequently, the dark region was displayed to cover half of the well for 10 min (LD condition) and each fish’s x-y coordinates were extracted via online processing at 10 frames per second (fps) and analyzed offline in Python (v3.9) and Microsoft Excel 365.

### Sleep and Locomotion Assay

The details of this sleep and locomotion assay were recently published and described in Basnakova et al. (2021). Based on the cited paper, we further added custom Python (v3.9) scripts for the current study. In short, larval zebrafish between the age of 6 and 9 dpf were plated individually in a 48-well plate. Each well of the plate was filled with 300 μL of E3 water or E3 of a specific salt concentration (50 or 100 mM). The recording was set to start from around 3 p.m. to 11 a.m. the next morning. During the recording time (20 h), 5 alternating light-dark (L-D) transitions, each lasting for 30 min from 5:30 to 9:30 p.m. were presented. Sleep data was calculated for the lights-off period from 11 p.m. to 9 a.m. Between 1 and 6 a.m. at night, their arousal threshold was measured by giving them a series of acoustic startle stimuli [for details see Basnakova et al. (2021)]. The x-y coordinates of each image were extracted via online processing at 5 fps and analyzed offline in Python and Microsoft Excel 365.

### Behavioral Analysis

As described in Basnakova et al. (2021), swim speed in both the L/D choice assay and sleep and locomotion assay was quantified by distance (mm) traveled per unit time (min). Sleep and rest scores were quantified by using a threshold of having less than 5 s of activity in a given minute. We also applied the conventional threshold of less than 1 s of activity in a given minute (Prober et al., 2006) with similar results (Supplementary Figure 2). In the L/D choice assay, freezing behavior in the light side was quantified by any 1-s bins with a distance moved smaller than 0.18 mm (roughly 1 pixel). Freezing ratio is thus determined by total freezing seconds divided by total duration. High-speed swims (escapes) in the light side were quantified by any 1-s bins with a distance moved larger than 9 mm (roughly 50 pixels or 2 body length of the larvae). An escape episode may span for more than 1 s, but in this case it is still counted as 1 escape episode. Freezing and escapes were not quantified in the sleep and locomotion setup.

Dark side ratio was quantified as the percentage of time spent in the dark/total time in the L/D choice assay. In both the L/D choice assay and sleep and locomotion assay, the thigmotaxis ratio of each fish from each frame was calculated by the distance of the fish’s x-y position to the center x-y point of the well. If the distance is within 0.71 of the radius of the active ROI region, then the fish’s location in that frame is classified as not in the wall area (i.e., in the center region). In contrast, if the distance is equal to or above 0.71 of the radius, the fish’s location is classified as in the vicinity of the wall area. A 1 versus 0.71 ratio of the radius gives two concentric circles such that the area of the inner circle is the same size as the outer circle (i.e., the vicinity of the wall area).

The L-D transition in the sleep and locomotion assay commenced at 5:30 p.m. and repeated for 5 cycles with each light or dark phase lasting for 30 min. As we observed habituation toward later transitions, only the first L-D transition data was analyzed to measure the true initial response from the fish to the first L-D transition. However no difference was observed when additional transitions were averaged.

Night time arousal threshold was determined by calculating the distance traveled of each fish within 5 frames (1 s) following the acoustic stimulus for at least 7 pixels (0.7 mm) from its original position [for details please see Basnakova et al. (2021) or Woods et al. (2014)]. We only analyzed the data from 1 to 2 a.m. because the fish were mostly not responding to the auditory stimuli after 3 a.m.

Fish behavioral data were excluded from further analysis if the fish did not survive the overnight salt exposure or their locomotion was extremely low (<50 mm/10 min) to determine thigmotaxis. In such cases, only 1 fish was excluded in the 50 mM group in the sleep data. In the L/D choice assay, 1 fish from the 0 mM group and 1 fish from the 50 mM group were excluded in the prolonged 40% condition in the L/D choice assay (Supplementary Table 1).

Live versions of the analysis code are maintained at https://github.com/CarolineWeeLab/EZBehavior.

### Salt Stress Feeding Assay

This protocol was adapted from Wee et al. (2019) and performed on 6–7 dpf larvae. Briefly, paramecia cultures were harvested the day before, then concentrated and labeled with 2.5 mg/ml lipid dye at the start of the experiment. Fishes were fed in the morning for 1 h 30 min and subsequently starved for 2 h in 10-cm petri dishes. Salt solutions were added 1 h 50 min after starvation in acute conditions, or from the start of the 2-h starvation period for the prolonged condition. Zebrafish were exposed to a final salt concentration of 50, 100, 150, 200, or 250 mM in feeding assays. After 10 min, 1 mL of concentrated paramecia was pipetted into each petri dish and fish were allowed to feed for 15 min. Fish were then rapidly fixed using 4% paraformaldehyde at 4°C overnight before washing in PBS and imaging.

### Feeding Assay Imaging and Data Analysis

The fixed zebrafish larvae samples were arranged on 35 mm dishes with a 14 mm microwell with a glass coverslip, taking care to ensure their guts were not in mutual contact. Brightfield and Cy5 images were captured using a Leica M205 FA fluorescence stereoscope. Brightfield exposure was set to 20 ms at 40% intensity while Cy5 exposure was set to 100 ms and 100% intensity. Images were acquired in a darkened room. Instance segmentation masks of the fish guts were generated using a custom-trained PointRend model. The mean fluorescence intensities inside the guts were obtained and normalized to the control for aggregation across repeats. The PointRend module enhances Mask R-CNN instance segmentation predictions by applying the concept of image rendering to produce finer predictions with higher resolution (Kirillov et al., 2019). Our training dataset comprised 86 brightfield images similar to Figure 4A each with approximately 25 instances of guts traced manually using Labelme (Wada, 2020) which served as ground truth labels. We applied transfer learning by using the Mask R-CNN model that was pre-trained on the COCO image dataset (Lin et al., 2014). PointRend was then trained in Google Colaboratory using the cloud-based GPU. On the test set, our model achieved a mean F1 score of 0.856. Live versions of the code are maintained at https://github.com/CarolineWeeLab/EZgut.

### Statistical Analysis

Each experiment was performed with at least two independent replicates of 6–18 fish per treatment for L/D choice assay or sleep and locomotion assays, and 3–4 replicates of 15–32 fish per treatment for feeding assays (detailed in Supplementary Table 1).

For two groups or multiple paired comparisons, we use estimation statistics (Ho et al., 2019) to provide effect sizes as Cohen’s *d* values as well as *p*-values from 5,000 bootstrap resampling permutation tests, unless specified otherwise. Data are plotted as mean ± 95% confidence interval (CI) while all effect size data are plotted as Cohen’s *d* value ± 95% confidence interval. Two-way ANOVA was conducted to study the interaction between light/dark status and salt stress on thigmotaxis behavior as well as between salt stress and genotype on feeding. Pearson’s correlation coefficient was calculated to study the relationships between various endophenotypes. We did not correct for multiple comparisons. Multiple *post hoc* Mann-Whitney *U* tests with Bonferroni correction were conducted to further examine this with respect to feeding. Spearman’s rank correlation coefficient was calculated to examine the monotonic relationship between salt stress and feeding. Asterisks indicate *p < 0.05, ^**^p < 0.01, and ^***^p < 0.001.

## Results

### Acute Salt Stress Elevates Thigmotaxis During Light/Dark Choice Behavior but Does Not Affect Dark Avoidance

As expected, fish in a L/D choice assay (Figure 1A) showed dark avoidance at both 80 and 40% transparencies (i.e., low and high-dark intensities), with stronger dark avoidance at high-dark intensities (Figure 1B). There was however no significant difference in the time spent on the light side across salt concentrations (0, 50, or 150 mM) at both dark intensities (Figure 1C). Notably, on the measure of thigmotaxis, there was a significant enhancement of the thigmotaxis ratios in the LD period for both 50 and 150 mM salt concentrations, but not in the all-light (AL) condition (Figures 1D,E). This effect occurred at both dark intensities (Figure 1E) but was more prominent at higher dark intensities (Figure 1D). Indeed, there was a significant interaction between salt concentrations and light/dark status (LD vs. AL) on the measure of thigmotaxis (Figure 1E, *p* = 0.047 for interaction in the 40% and *p* = 0.028 for the 80%, two-way mixed ANOVA).

In terms of swim speed, no differences were observed in the AL period (Figures 1F,G), except for a moderate increase in swim speeds at low-salt (50 mM) and low-dark (80%) intensities. During the LD period (Figure 1G), high (150 mM) salt significantly reduced the total distance traveled per unit time at high-dark (40%) intensities. When we quantified freezing behavior, we found that there was a significant, salt dose-dependent increase in freezing that was potentiated by LD conditions (Figures 1H,I). The frequency of high-speed swims (which we define as escapes, see section “Materials and Methods”) was also moderately enhanced by both salt concentrations in the high-dark (40%) condition, and by high salt (150 mM) in the low-dark condition (80%) (Supplementary Figures 1A,B). Importantly, freezing or escape rates were generally not significantly different either at the center or periphery of the wells hence these behaviors cannot explain the enhanced thigmotaxis [*p* > 0.05 for freezing and escapes at center vs. wall areas, except for freezing wall < freezing center (*p* = 0.034 in acute 0 mM, AL, 40% transparency) and escapes wall > escapes center (*p* = 0.0016 in prolonged 0 mM, LD, 80% transparency and *p* < 0.0001 in prolonged 50 mM, LD, 80% transparency)].

To identify associations between the endophenotypes observed, Pearson’s correlation coefficients were quantified between each individual fish’s dark avoidance, thigmotaxis, locomotion, freezing, and escape during AL and LD conditions, for all salt concentrations and dark intensities (Figure 1J). Overall, the correlations between these endophenotypes were weaker at high-dark (40%, right) as compared to low-dark (80%, left) intensities. There were expected negative correlations between swim speed and freezing, and positive correlations between swim speed and escapes, which were clearest at baseline (0 mM) salt conditions at low-dark (80%) intensities. Dark ratio was weakly correlated with thigmotaxis, and significant only at some salt and dark intensities. Across salt concentrations, there were also positive correlations between thigmotaxis, swim speed, and escapes, which seemed to be abolished at high-dark (40%) intensities. At high (150 mM) salt concentrations in LD conditions, a significant negative correlation between freezing and escapes emerged, across both dark intensities.

### Prolonged Salt Stress Increases Dark Avoidance but Not Thigmotaxis

Next, we examined the effects of prolonged (2–4 h) rather than acute salt exposure on L/D choice, thigmotaxis, and other endophenotypes (Figure 2A). Since 150 mM exposure to salt was found to be lethal after several hours, the maximum concentration utilized was 100 mM. Interestingly, 100 mM salt did enhance dark avoidance at low-dark (80%) intensities, which was accompanied by a significant reduction in swim speed (Figures 2C,G). High-dark (40%) intensities already induced strong dark avoidance in controls, hence likely imposing a ceiling effect (Figures 2B,C). Surprisingly, there was no longer a significant effect on thigmotaxis even in the LD context (Figures 2D,E). The weaker effects on thigmotaxis could potentially be due to the overall higher baseline thigmotaxis ratios (even at 0 mM salt, compared with Figures 1D,E), for reasons such as batch-to-batch differences or the pre-exposure protocol. 100 mM salt reduced locomotion (Figures 2F,G) and enhanced freezing (Figures 2H,I) particularly in the LD context at low-dark (80%) intensities. The absence of effects at high-dark (40%) intensities are likely because baseline freezing rates were already high in the latter, even in the absence of salt. There were no significant effects of prolonged salt stress on escapes (Supplementary Figures 1C,D).

We observed similar associations between endophenotypes (e.g., between thigmotaxis and swim speed, swim speed with freezing or escapes) in prolonged as with acute salt stress conditions (Figure 2J). Again, the correlations were weaker at high-dark (40%) intensities. Upon prolonged salt exposure, dark ratio was more strongly correlated with thigmotaxis at low-dark (80%) intensities, as compared to acute exposure. At prolonged high-salt (100 mM) exposure, escapes again became negatively correlated with freezing, similar to acute conditions.

### Salt Stress Reduces Sleep With Minimal Effects on Other Behaviors

**FIGURE 3:**
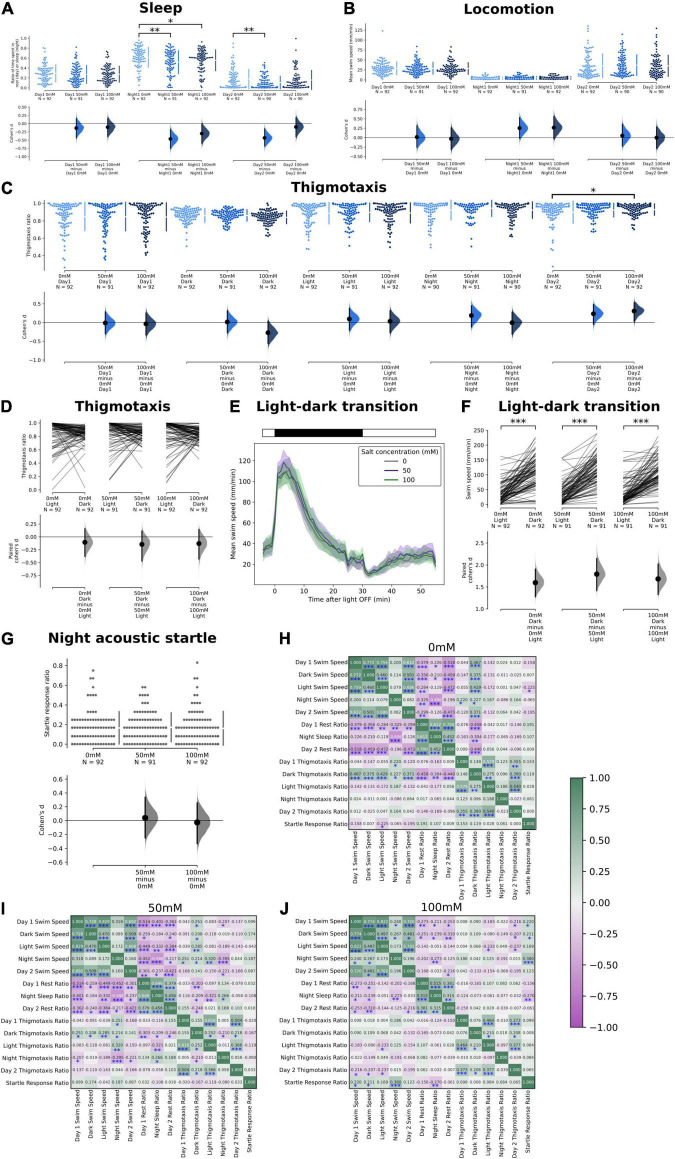
Effects of salt stress on thigmotaxis and locomotion in the sleep and locomotion assay. **(A)** Ratio of time spent in rest during Day 1, during sleep in Night 1, and during Day 2. A significant reduction in the ratio of time spent resting was observed at night at both 50 mM (*p* = 0.0018) and 100 mM salt concentrations (*p* = 0.0454). There was also a reduction in the ratio of time spent resting in Day 2 at 50 mM salt concentration (*p* = 0.0034). **(B)** Mean swim speed during Day 1, during sleep in Night 1, and during Day 2. No significant changes were observed. **(C)** Thigmotaxis ratio during Day 1, Dark Period, Light Period, Night 1, and Day 2 where a significant increase in thigmotaxis was observed in Day 2 at 100 mM salt concentration (*p* = 0.035). **(D)** Change of thigmotaxis ratio during the first light to dark transition period. No significant changes were observed. **(E)** Swim speed as a function of time during the first light to dark transition period. **(F)** Comparisons of mean swim speed before and during the dark transition period. There was a significant increase in swim speed in the dark period at all concentrations (*p* < 0.0001). **(G)** There was no significant change in the startle response ratio of the three strongest acoustic stimulus intensities (100, 84, and 72%) during the first 2 h after midnight. **(H–J)** Correlation coefficient (*r*) of the swim speed, rest/sleep ratio, thigmotaxis ratio, and startle response ratio at different salt concentrations [0 mM in **(H)**, 50 mM in **(I)**, and 100 mM in **(J)**] (*p < 0.05, ^**^p < 0.01, and ^***^p < 0.001).

Next, on a different high-throughput (48-well) behavioral setup, we quantified the effects of 50 or 100 mM prolonged salt exposure on different behaviors. Sleep time was represented as the percentage of time spent in rest (during Day 1 and 2) and in sleep (during Night 1) (Figure 3A and Supplementary Figure 2). Whereas salt exposure did not affect their rest during Day 1, sleep time was significantly reduced across both salt concentrations during the night, and was also reduced on Day 2 for the low-salt (50 mM) condition (Figure 3A and Supplementary Figure 2). Mean swim speed of the fish was quantified across the entire recording period (Day 1, Night 1, and Day 2) as a function of salt concentrations (Figure 3B). There was no significant change in mean swim speed, though there was a trend toward increased swim speed at night, which may be associated with the reduced sleep observed during the same period (Figure 3B).

**FIGURE 4:**
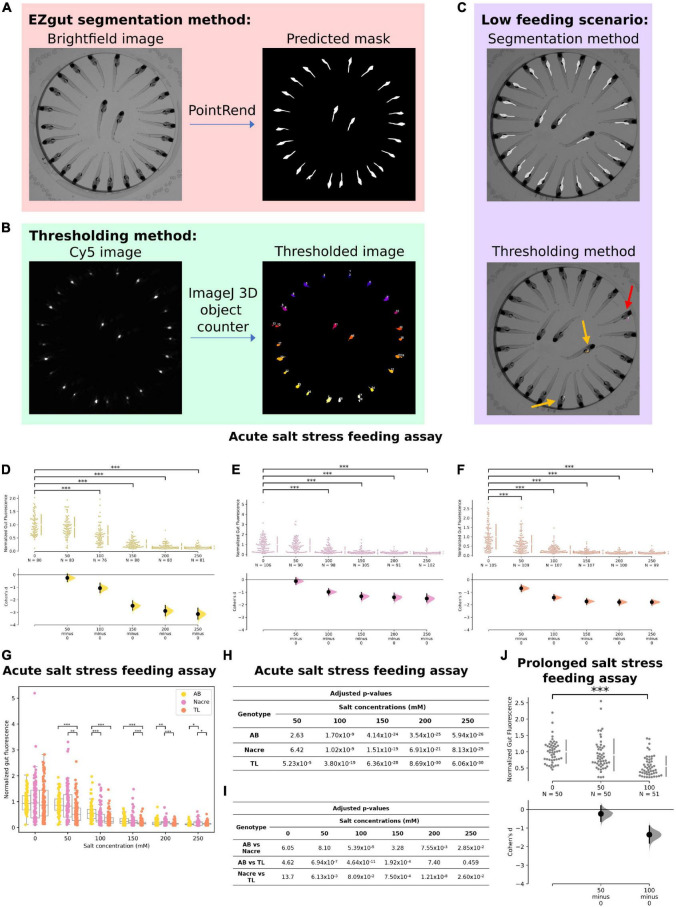
Effect of osmotic stress on feeding. **(A)** In our new analysis method, EZgut, the PointRend model generates gut segmentation masks from brightfield images of zebrafish larvae. Gut fluorescence can be quantified by the pixel intensities of the corresponding Cy5 image [see panel **(B)**] that fall within each distinct gut in the mask. **(B)** The thresholding method requires the user to choose a threshold based on the Cy5 image for the control sample which is then applied to all other Cy5 images for that experiment. The method uses the 3D objects counter tool in ImageJ. **(C)** An example of a low feeding scenario where the thresholding method results in loss of data where only two guts were identified (yellow arrows) with 1 false positive where fluorescent debris was detected outside the gut (red arrow). However, the segmentation method still can detect all the guts. **(D–F)** Salt stress causes significant reduction in feeding in WTAB, Nacre and TL zebrafish larvae (*p < 0.05, ^**^p < 0.01, and ^***^p < 0.001). **(G)** Both genotype and salt concentration interact to cause reduction in feeding due to salt stress. **(H,I)** Table of *p*-values for **(D–F)** and **(G),** respectively. **(J)** After prolonged exposure (2 h), a significant reduction in feeding was observed at 100 mM (*p* = 3.36 × 10^–9^) salt concentration but not at 50 mM (*p* = 0.575) in wild-type AB larvae.

We also quantified the thigmotaxis ratios in this 48-well setup across different time periods, including during the dark and light periods of the first L-D transition, as a function of salt concentrations. We did not observe any changes in thigmotaxis except on Day 2, where thigmotaxis was significantly increased in the 100 mM condition (Figures 3C,D). Hence, the heightened activity induced by L-D transitions (Figures 3E,F) did not generally correlate with higher thigmotaxis (Figures 3C,D). The lack of effect on thigmotaxis could again potentially be explained by a higher baseline thigmotaxis rate in this smaller 48-well setup, even for the no-salt controls, consistent with observations in the literature (Schnörr et al., 2012). Similarly, there was no difference in L-D transition behavior across salt concentrations (Figures 3E,F). The arousal threshold (response to auditory stimuli during the early hours after midnight) was also comparable among the fish treated with different salt concentrations (Figure 3G). Hence, the reduced night-time sleep in 50 and 100 mM salt did not correlate with differences in arousal threshold.

Finally, we cross-correlated each individual fish’s behavioral output on the above measures (swim speed, sleep time, thigmotaxis, and startle) across different time periods (Figures 3H–J). In the control conditions, there were expected positive correlations between the same measures at different time points, and negative correlations between swim speeds and sleep/rest time. Interestingly we observed correlations between thigmotaxis in the dark (during L-D transition) and swim speeds or sleep time at various time periods, which could be consistent with the positive correlations between thigmotaxis and swim speeds we observed in the L/D choice assay. Notably, the correlations between these different measures decreased with increasing salt concentrations.

### Salt Stress Reduces Feeding in a Novel Gut Fluorescence Assay

Finally, we used a published gut fluorescence-based feeding assay to examine the effect of osmotic stress on feeding. We improved on this previous assay by implementing machine learning-based automatic instance segmentation for gut fluorescence analysis (Figure 4A) instead of the previous thresholding protocol (Figure 4B) (Wee et al., 2019). The benefits of this new method are 2-fold. First, by eliminating the step of thresholding the fluorescence image, we have reduced subjectivity in the analysis method. Second, since the same threshold must be used consistently for all conditions within one experiment, the thresholding protocol can result in loss of data in low feeding conditions, which may in extreme cases skew the interpretation of results (Figure 4C). This data loss does not occur with the gut segmentation method as segmentation occurs independently of the fluorescence images.

When acutely exposed to increasing concentrations of salt, we observed a significant reduction in feeding at salt concentrations of 100 mM and above for wild-type AB genotype larvae (Figure 4D). This data confirms that the gut fluorescence feeding assay can be integrated with osmotic stress to quantify stress-induced anorexia. Similar trends were observed for the salt stress feeding assay conducted on Nacre and TL larvae (Figures 4E,F) although the strongest negative correlation and effect size was observed for the AB genotype. There was a significant interaction between genotype and salt concentration and significant differences between AB, Nacre and TL genotypes at multiple concentrations (adjusted *p*-value < 0.05, multiple Mann-Whitney *U* tests with Bonferroni correction) (Figures 4G–I).

We also examined the effects of prolonged (2-h) salt stress on feeding, and observed a similar suppressive effect at higher (100 mM) concentrations in wild-type AB larvae (Figure 4J). While we did not examine locomotor behavior in this feeding assay, our L/D choice assay (Figures 1, 2) suggest minimal effects of up to 150 mM acute or 100 mM prolonged salt exposure on locomotor behavior in AL contexts, also consistent with De Marco et al. (2014). Prolonged salt of up to 100 mM similarly did not affect Day 1 rest or swim speed in our sleep and locomotion assay (Figure 3).

## Discussion

In this study we examined the effects of osmotic stress on a range of anxiety endophenotypes, and found that they are differentially affected by this stressor in an intensity and duration-dependent manner. Whereas feeding was similarly suppressed by acute and prolonged stress, dark avoidance was only enhanced after longer (2–4 h) exposure to osmotic stress. The differences between acute and prolonged salt exposure could potentially be due to the activation of secondary stress responses, including transcriptional changes, or osmoregulatory adaptations (Barton, 2002). Based on the literature, we expect cortisol levels to fall from peak levels but remain elevated after longer exposure, which may also explain some differences in behavioral effects (Liebert and Schreck, 2006; Kammerer et al., 2010; De Marco et al., 2014). An important consideration in using osmotic stress is that salt not only triggers stress-related behavioral changes, but also poses a survival threat to larval zebrafish, as evident from our observations that longer term exposure at higher salt concentrations (>100 mM) is lethal to larval zebrafish. Hence, some aspects of behavior after prolonged salt exposure might be explained by the biologically harmful effects of salt to freshwater fish.

Further, the manifestation of some of these endophenotypes is context-dependent, such as in the case of thigmotaxis (and freezing) and the presence of a dark zone. This dark-induced potentiation of thigmotaxis is likely due to a heightened anxiety state, but we do not simultaneously observe enhanced dark avoidance behavior. Notably, a weaker effect on thigmotaxis was seen after prolonged salt exposure, though the effect could have been obscured by the generally-elevated thigmotaxis levels we observed under all conditions (including no-salt controls). Except for the 100 mM salt concentration on Day 2, we also did not observe any significant effects of salt on thigmotaxis in our sleep and locomotion 48-well arena, both in complete light or in darkness. It is also possible that factors such as arena size (6-well vs. 48-well) could have influenced the expression of the endophenotype, especially since the fish in the sleep and locomotion assay, including no-salt controls, already had higher baseline thigmotaxis. These are also not directly comparable assays in terms of the time of salt exposure, with the L/D choice assay being for 2–4 h and the sleep and locomotion assay being performed overnight.

As expected, locomotor endophenotypes such as swim speed, freezing, escapes, and sleep showed stronger positive or negative correlations with each other. Further, in certain contexts, swim speed, thigmotaxis, and dark avoidance were positively-correlated; however they were also dissociable, especially at higher dark or salt concentrations. Overall, it appears that increased stressor intensities weaken associations between endophenotypes, which could possibly be explained by more erratic or extreme behavior patterns. It would be interesting to determine how these endophenotype decorrelations are reflected in brain activity changes. Our results also complement previous reports of dissociations between anxiety endophenotypes (Blaser and Rosemberg, 2012; Bai et al., 2016; van den Bos et al., 2017; Golla et al., 2020).

The relationship between stress and feeding has been established in humans, rodents and zebrafish (Born et al., 2010; Rudenga et al., 2013; De Marco et al., 2014; Weidenfeld and Ovadia, 2017). Stress results in appetite dysregulation, causing stress-induced anorexia or in other cases, excessive caloric intake. In zebrafish, salt stress has been shown to reduce food preference via activation of the HPA axis and subsequent cortisol release (De Marco et al., 2014). Isolation stress-induced anorexia has also recently been reported (Wee et al., 2022). The existing method of quantifying stress-induced anorexia requires custom-built arenas and video tracking methods (De Marco et al., 2014), rendering the assay relatively low-throughput and complicated to analyze. We have adapted a gut fluorescence feeding assay (Wee et al., 2019, 2021) into a salt stress feeding assay that is high-throughput and scalable. We have also enhanced the gut fluorescence quantification with machine learning-based instance segmentation of zebrafish larval guts.

We report a similar amount of stress-induced anorexia after both acute and prolonged salt stress. Notably, we also observed a difference in acute salt stress-induced anorexia across different genotypes. While we did not examine the effects of genotype on the other behavioral measures, there exists evidence in the literature of genotype differences, particularly between AB and TL larvae, in L-D transitions (AB stronger than TL) and startle habituation (AB more than TL), as well as baseline HPA axis activity (AB higher than TL) (van den Bos et al., 2017). These results emphasize the importance of considering zebrafish genotype in the analysis of anxiety behavior as strains differ in their sensitivity to stressors (van den Bos et al., 2017).

We also note that, as a caveat of all behavioral assays shown, there could be alternative explanations to some of the endophenotypes we observed (e.g., reduced sleep, enhanced thigmotaxis, or reduced feeding) that may not necessarily reflect anxiety. For example, seizures can induce thigmotactic behavior, and could potentially be induced by high osmotic stress, though the specificity of our observed phenotypes (e.g., no overall change in swim speed, specificity of thigmotaxis to LD conditions) argues against this. Similarly, a reduction in feeding or enhanced movements at night could be affected by purely sensorimotor deficits or changes, without invoking anxiety. In fact, these arguments emphasize another benefit of examining multiple endophenotypes in parallel – to help rule out possible confounds that could explain observed behaviors. Finally, it would be interesting to examine how social avoidance or orienting endophenotypes are affected by osmotic stress, or relate to other group behaviors such as feeding, since they were not analyzed in this study (Tan et al., 2022).

A challenge in testing for multiple endophenotypes is that the different assays often do not have standard arena sizes or experimental setups. This could result in batch effects or experimental differences that reduce the comparability of the results, not to mention across different labs. While standardization is theoretically possible, compromises would have to be made on throughput, ease and cost. Despite these constraints, our results suggest that examining a range of endophenotypes paints a more well-rounded picture of anxiety behavior.

In conclusion, we have shown specific effects of osmotic stress on thigmotaxis, sleep, and feeding, which are influenced by factors such as stressor intensity, light or dark status, and genotype. We found that the correlations and dissociations between anxiety endophenotypes were context- and stressor intensity-dependent. Interestingly, increased salt stress (and dark intensities in the L/D choice assay) led to an observed decorrelation of some endophenotypes. Our results illustrate the many-to-many relationship between endophenotypes, behaviors, and underlying biological mechanisms. Therefore, cross-examination of endophenotypes via multiple behavioral assays is important to achieve a holistic understanding of stress and anxiety behaviors and their underlying mechanisms (Tan et al., 2022). There may be valuable insights drawn from the correlations and dissociations of endophenotypes that we can exploit in therapeutic approaches to treating stress-related mental disorders.

## Data Availability Statement

The original contributions presented in this study are included in the article/Supplementary Material, further inquiries can be directed to the corresponding author.

## Ethics Statement

The animal study was reviewed and approved by A*STAR Biomedical Research Council Institutional Animal Care and Use Committee (IACUC).

## Author Contributions

CW, R-KC, and JT conceived of the project, designed the experiments, and wrote and edited the manuscript. R-KC developed and optimized hardware, software, and protocols for the light/dark choice and sleep and locomotion assay. JT developed and validated the EZgut analysis platform. KXC performed all experiments. CT and CW helped develop and optimize the behavioral assays. R-KC, JT, and KXC analyzed data for the manuscript. All authors contributed to the article and approved the submitted version.

## Conflict of Interest

The authors declare that the research was conducted in the absence of any commercial or financial relationships that could be construed as a potential conflict of interest.

## Publisher’s Note

All claims expressed in this article are solely those of the authors and do not necessarily represent those of their affiliated organizations, or those of the publisher, the editors and the reviewers. Any product that may be evaluated in this article, or claim that may be made by its manufacturer, is not guaranteed or endorsed by the publisher.
