# Supplementary material for: Osmotic Stress Uncovers Correlations and Dissociations Between Larval Zebrafish Anxiety Endophenotypes
Source: Front Mol Neurosci. 2022 Jun 24;15:900223. doi: 10.3389/fnmol.2022.900223 (PMC9269111; doi:10.3389/fnmol.2022.900223)
Supplement: Supplementary file 1 [file Data_Sheet_1.pdf]

Supplementary Figures and Legends

Supplementary Figure 1: Effect of osmotic stress on high-speed movements (escapes). (A) At 80% transparency, the larvae displayed higher escape frequency in the LD compared to AL condition at 150 mM salt concentration ($p = 0.0116$) but not at other concentrations (0 mM: $p = 0.384$, 50 mM: $p = 0.202$). At 40% transparency, higher escape frequency in LD was observed at 0 mM salt concentration ($p = 0.0052$) but not at higher concentrations (50 mM: 0.0582, 150 mM: 0.405). **(B)** At 80% transparency in the LD condition, the addition of 150 mM of salt caused a significant increase in escape frequency ($p = 0.0244$) compared with the control group. At 40% transparency, the increase in escape frequency was only observed in the AL condition but at all salt concentrations (50 mM vs 0 mM: $p = 0.0492$, 150 mM vs 0 mM: $p = 0.022$). **(C, D)** No effect was observed on escape frequencies in the prolonged salt stress condition across all salt concentrations, darkness levels and AL vs LD conditions. For all plots, * $p < 0.05$, ** $p < 0.01$, *** $p < 0.001$.

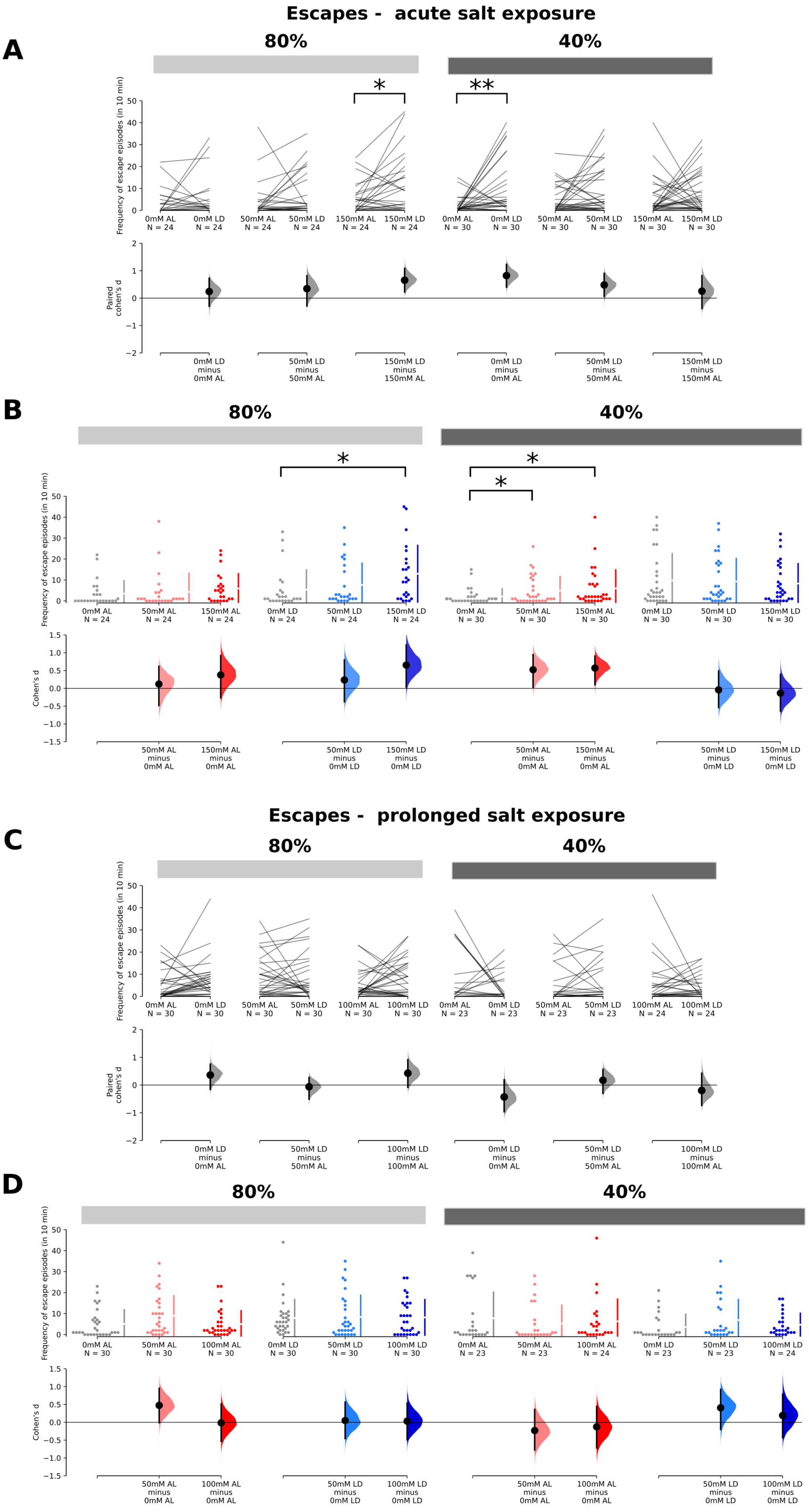
Escapes - acute salt exposure
A
80%
40%
Frequency of escape episodes (in 10 min)
0mM AL N = 24
0mM LD N = 24
50mM AL N = 24
50mM LD N = 24
150mM AL N = 24
150mM LD N = 24
0mM AL N = 30
0mM LD N = 30
50mM AL N = 30
50mM LD N = 30
150mM AL N = 30
150mM LD N = 30
Paired cohen's d
0mM LD minus 0mM AL
50mM LD minus 50mM AL
150mM LD minus 150mM AL
B
Cohen's d
50mM AL minus 0mM AL
150mM AL minus 0mM AL
50mM LD minus 0mM LD
150mM LD minus 0mM LD
Escapes - prolonged salt exposure
C
100mM AL N = 30
100mM LD N = 30
0mM AL N = 23
0mM LD N = 23
50mM AL N = 23
50mM LD N = 23
100mM AL N = 24
100mM LD N = 24
100mM LD minus 100mM AL
D
100mM AL minus 0mM AL
100mM LD minus 0mM LD

Supplementary Figure 2: Effect of osmotic stress on sleep. (A, B, C) defines a minute of sleep as less than 1 s of movement per minute while **(D, E, F)** less stringently defines a minute of sleep as less than 5 s of movement per minute, which is the criterion we used for Fig. 3. **(A, D)** Using both criteria, a significant reduction in average sleep bout length was observed at 50 mM salt concentration (1 s: $p = 0.013$, 5 s: $p = 0.002$) but not at 100 mM (1 s: $p = 0.703$, 5 s: 0.0726). **(B, E)** There was no significant change in the sleep bout frequency at all salt concentrations. **(C, F)** A significant reduction in the ratio of time spent in sleep was detected at 50 mM salt concentration (1 s: $p = 0.011$, 5 s: $p = 0.0018$) using both criteria, while at 100 mM, a significant reduction was only detected when using the 5 s criterion (1 s: $p = 0.324$, 5 s: $p = 0.0454$). **(G)** Lower panel shows the averaged sleep trace of the zebrafish larvae across day 1 (L-D transition experiment), night (sleep) and day 2. Representative raw traces of larvae in each salt concentration is shown above (0 mM in black, 100 mM in red) where apart from the night phase, both exhibit similar behavior in the L-D transition experiment and in the day. For all plots, * $p < 0.05$, ** $p < 0.01$, *** $p < 0.001$.

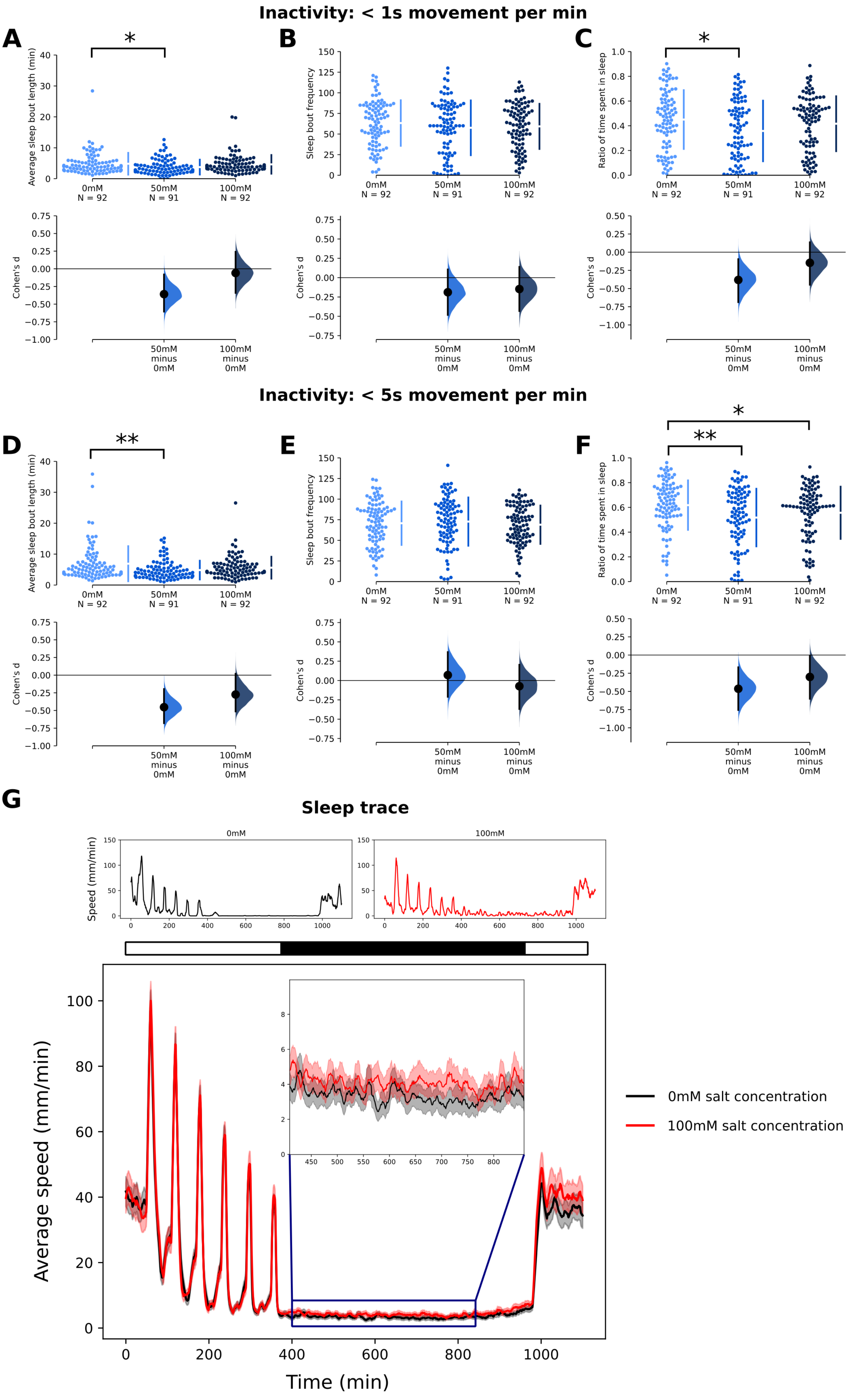
Inactivity: < 1s movement per min
A
B
C
Average sleep bout length (min)
Sleep bout frequency
Ratio of time spent in sleep
0mM
N = 92
50mM
N = 91
100mM
N = 92
Cohen's d
50mM
minus
0mM
100mM
minus
0mM
Inactivity: < 5s movement per min
D
E
F
G
Sleep trace
0mM
100mM
Speed (mm/min)
Average speed (mm/min)
Time (min)
0mM salt concentration
100mM salt concentration
