# Supplementary material for: Osmotic Stress Uncovers Correlations and Dissociations Between Larval Zebrafish Anxiety Endophenotypes
Source: Front Mol Neurosci. 2022 Jun 24;15:900223. doi: 10.3389/fnmol.2022.900223 (PMC9269111; doi:10.3389/fnmol.2022.900223)
Supplement: Supplementary file 2 [file Table_1.docx]

Supplementary Table 1: Summary of experiments performed

| **Experiment information** | | | | **Experiment sample sizes**  **(number of larvae)** | | | | | | **Exclusions during analysis** |
| --- | --- | --- | --- | --- | --- | --- | --- | --- | --- | --- |
| **Assay** | **Treatment** | **Strain** | **Salt Conc.**  **(mM)** | **1** | **2** | **3** | **4** | **5** | **6** |  |
| Light-Dark  (40%) | Acute | AB | 0 | 18 | 12 | - | - | - | - | 0 |
|  |  |  | 50 | 18 | 12 | - | - | - | - | 0 |
|  |  |  | 150 | 18 | 12 | - | - | - | - | 0 |
| Light-Dark  (40%) | Prolonged | AB | 0 | 6 | 18 | - | - | - | - | 1 |
|  |  |  | 50 | 6 | 18 | - | - | - | - | 1 |
|  |  |  | 100 | 6 | 18 | - | - | - | - | 0 |
| Light-Dark  (80%) | Acute | AB | 0 | 6 | 18 | - | - | - | - | 0 |
|  |  |  | 50 | 6 | 18 | - | - | - | - | 0 |
|  |  |  | 150 | 6 | 18 | - | - | - | - | 0 |
| Light-Dark  (80%) | Prolonged | AB | 0 | 12 | 12 | 6 | - | - | - | 0 |
|  |  |  | 50 | 12 | 12 | 6 | - | - | - | 0 |
|  |  |  | 100 | 12 | 12 | 6 | - | - | - | 0 |
| Sleep / Locomotion | Prolonged | AB | 0 | 16 | 16 | 12 | 16 | 16 | 16 | 0 |
|  |  |  | 50 | 16 | 16 | 12 | 16 | 16 | 16 | 1 |
|  |  |  | 100 | 16 | 16 | 12 | 16 | 16 | 16 | 0 |
| Feeding | Acute | AB | 0 | 30 | 25 | 25 | - | - | - | 0 |
|  |  |  | 50 | 31 | 25 | 27 | - | - | - | 0 |
|  |  |  | 100 | 31 | 20 | 25 | - | - | - | 0 |
|  |  |  | 150 | 31 | 24 | 25 | - | - | - | 0 |
|  |  |  | 200 | 31 | 25 | 24 | - | - | - | 0 |
|  |  |  | 250 | 29 | 27 | 25 | - | - | - | 0 |
|  |  | TL | 0 | 25 | 22 | 26 | 32 | - | - | 0 |
|  |  |  | 50 | 25 | 25 | 26 | 34 | - | - | 0 |
|  |  |  | 100 | 26 | 25 | 24 | 32 | - | - | 0 |
|  |  |  | 150 | 26 | 25 | 25 | 31 | - | - | 0 |
|  |  |  | 200 | 24 | 22 | 24 | 30 | - | - | 0 |
|  |  |  | 250 | 25 | 24 | 23 | 27 | - | - | 0 |
|  |  | Nacre | 0 | 32 | 17 | 26 | 31 | - | - | 0 |
|  |  |  | 50 | 22 | 16 | 26 | 26 | - | - | 0 |
|  |  |  | 100 | 27 | 20 | 26 | 25 | - | - | 0 |
|  |  |  | 150 | 27 | 26 | 28 | 24 | - | - | 0 |
|  |  |  | 200 | 20 | 16 | 29 | 26 | - | - | 0 |
|  |  |  | 250 | 33 | 15 | 23 | 31 | - | - | 0 |
|  | Prolonged | AB | 0 | 25 | 25 | - | - | - | - | 0 |
|  |  |  | 50 | 25 | 25 | - | - | - | - | 0 |
|  |  |  | 100 | 26 | 25 | - | - | - | - | 0 |
